# In-vivo neuronal dysfunction by Aβ and tau overlaps with brain-wide inflammatory mechanisms in Alzheimer’s disease

**DOI:** 10.3389/fnagi.2024.1383163

**Published:** 2024-06-19

**Authors:** Lazaro M. Sanchez-Rodriguez, Ahmed F. Khan, Quadri Adewale, Gleb Bezgin, Joseph Therriault, Jaime Fernandez-Arias, Stijn Servaes, Nesrine Rahmouni, Cécile Tissot, Jenna Stevenson, Hongxiu Jiang, Xiaoqian Chai, Felix Carbonell, Pedro Rosa-Neto, Yasser Iturria-Medina

**Affiliations:** ^1^Department of Neurology and Neurosurgery, McGill University, Montreal, QC, Canada; ^2^McConnell Brain Imaging Centre, Montreal Neurological Institute, Montreal, QC, Canada; ^3^Ludmer Centre for Neuroinformatics and Mental Health, Montreal, QC, Canada; ^4^McGill University Research Centre for Studies in Aging, Douglas Research Centre, Montreal, QC, Canada; ^5^Lawrence Berkeley National Laboratory, Berkeley, CA, United States; ^6^Biospective Inc., Montreal, QC, Canada

**Keywords:** Alzheimer’s disease, neuronal dysfunctions and alterations, whole-brain modeling, transcriptomics, amyloid – beta, tau and phospho-tau protein, inflammation, computational drug repurposing

## Abstract

The molecular mechanisms underlying neuronal dysfunction in Alzheimer’s disease (AD) remain uncharacterized. Here, we identify genes, molecular pathways and cellular components associated with whole-brain dysregulation caused by amyloid-beta (Aβ) and tau deposits in the living human brain. We obtained *in-vivo* resting-state functional MRI (rs-fMRI), Aβ- and tau-PET for 47 cognitively unimpaired and 16 AD participants from the Translational Biomarkers in Aging and Dementia cohort. Adverse neuronal activity impacts by Aβ and tau were quantified with personalized dynamical models by fitting pathology-mediated computational signals to the participant’s real rs-fMRIs. Then, we detected robust brain-wide associations between the spatial profiles of Aβ-tau impacts and gene expression in the neurotypical transcriptome (Allen Human Brain Atlas). Within the obtained distinctive signature of *in-vivo* neuronal dysfunction, several genes have prominent roles in microglial activation and in interactions with Aβ and tau. Moreover, cellular vulnerability estimations revealed strong association of microglial expression patterns with Aβ and tau’s synergistic impact on neuronal activity (*q* < 0.001). These results further support the central role of the immune system and neuroinflammatory pathways in AD pathogenesis. Neuronal dysregulation by AD pathologies also associated with neurotypical synaptic and developmental processes. In addition, we identified drug candidates from the vast LINCS library to halt or reduce the observed Aβ-tau effects on neuronal activity. Top-ranked pharmacological interventions target inflammatory, cancer and cardiovascular pathways, including specific medications undergoing clinical evaluation in AD. Our findings, based on the examination of molecular-pathological-functional interactions in humans, may accelerate the process of bringing effective therapies into clinical practice.

## Introduction

Neuronal dysfunction in Alzheimer’s disease (AD) is associated with toxic protein accumulation, including amyloid beta (Aβ) plaques and tau neurofibrillary tangles (NFTs) (Jack et al., 2018; Maestú et al., 2021). *In-vivo* animal experiments and modeling approaches support that Aβ and tau synergistically interact to impair brain function (Maestú et al., 2021; Targa Dias Anastacio et al., 2022; van Nifterick et al., 2022), inducing network hyperactivity as the disease progresses (Vossel et al., 2017; Busche and Hyman, 2020; Tok et al., 2022). However, when studying the disease and its biological basis in the living human brain, we continue to have critical limitations to concurrently measure neuronal activity, pathological severity, and molecular profiles. This issue represents a major obstacle to understanding the complex biological mechanisms underlying neuronal dysfunction in AD (Calabrò et al., 2021; Maestú et al., 2021; Iturria-Medina et al., 2022; Morgan et al., 2022; Nandi et al., 2022; Gabitto et al., 2023) and may have directly contributed to the limited efficacy of some proposed therapeutics (Iturria-Medina et al., 2018; Cummings et al., 2021).

Groundbreaking integrative computational modeling of *in-vivo* human pathophysiological processes offers a powerful alternative to overcome experimental shortcomings in AD research (Sotero and Trujillo-Barreto, 2008; Carbonell et al., 2018; Deco et al., 2018; Sanchez-Rodriguez et al., 2018; Stefanovski et al., 2019; Adewale et al., 2021; Iturria-Medina et al., 2021, 2022; Khan et al., 2022; Lenglos et al., 2022). Specifically, the scarcity of *in-vivo* recordings capturing the profound functional impacts of the disease’s neuropathological factors may be solved through data-informed mechanistic investigations. We recently proposed personalized computational models to estimate synergistic Aβ and tau effects on neuronal excitability in AD progression (Sanchez-Rodriguez et al., 2024). This method allowed us to robustly infer *in-vivo* patient-specific values of neuronal excitability and describe their associations with pathological severity, disease biomarkers (e.g., p-tau217, p-tau231) (Zetterberg and Blennow, 2021) and altered electroencephalographic indexes (Babiloni et al., 2013; Sanchez-Rodriguez et al., 2018). The obtained Aβ and tau functional weights effectively predicted cognitive decline in the AD-related cohort. Additionally, we demonstrated that Aβ and tau neurofunctional effects are spatially heterogeneous and significant at specific brain regions with consistent grey matter alterations in AD (Wang et al., 2015). On the other hand, and despite recent progress in characterizing post-mortem molecular profiles across multiple brain areas in AD cohorts (Gabitto et al., 2023; Ng et al., 2023), complete genetic spatial mapping of AD is lacking. As an alternative, computational approaches (Mullins and Kapogiannis, 2022; Ye et al., 2022; Tang et al., 2024) test for spatial correspondence between neuroimaging-derived indicators and the available genetic maps, notably the adult human brain transcriptome obtained by the Allen Brain Institute (Hawrylycz et al., 2012). Thus, in this study we sought to identify the genes, pathways, and cellular mechanisms underlying the effects of AD pathologies on human *in-vivo* neuronal activity throughout the entire brain.

We extend previous *in-vivo* AD pathophysiological studies in four fundamental ways. First, we utilize generative brain models to estimate the combined spatiotemporal influence of Aβ and tau (measured via PET) on neuronal activity (measured through fMRI biomarkers) for cognitively unimpaired and AD participants. Second, we use whole-brain transcriptomics to identify genes with spatial expressions that overlap with the regional neuronal activity effects of Aβ, tau, and their synergistic interaction. This analysis results in a clear and consistent Aβ + tau → neuronal-activity molecular signature, with both distinctive mechanisms and processes shared with diseases such as infection, cancer and retinal conditions. Major associations with the immune system, cell communication and developmental mechanisms exist, driven by the synergistic interaction of Aβ and tau. Third, we detect the cell types that are most likely related to neuronal activity alterations by the combined causal roles of Aβ and tau pathologies, observing a predominant role of microglia. Fourth, focusing on targeting functional pathways impaired by AD pathologies, we discover potential pharmacological interventions (from a small molecules library) modifying these diseased biological processes. This pioneering study, proposing a comprehensive examination of *in-vivo* neuronal dysregulation induced by AD pathology in humans, uncovers a multifaceted interplay between molecular signatures and functional mechanics associated with AD progression. It also supports the extended value of holistic computational approaches considering the critical tripartite relationship (molecular-pathological-functional) –rather than isolated disease components– thus offering new avenues for identifying effective therapeutic targets in neurodegeneration.

## Materials and methods

### Participants

Data was collected under the Translational Biomarkers in Aging and Dementia (TRIAD) cohort (https://triad.tnl-mcgill.com/). The study was approved by the McGill University PET Working Committee and the Douglas Mental Institute Research Ethics Boards and all participants gave written consent. We selected baseline assessments for 47 “cognitively unimpaired” and 16 “Alzheimer’s disease” subjects (Supplementary Table 1) according to clinical and pathophysiological diagnoses. All subjects underwent T1-weighted structural MRI, resting-state fMRI, Aβ (^18^F-NAV4694)- and tau (^18^F-MK-6240)- PET scans –see below and the provided references for processing details. The selected CU individuals were both Aβ and tau-negative while the AD subjects presented positive Aβ status (as determined visually by consensus of two neurologists blinded to the diagnosis) and cortical tau involvement (Braak et al., 1995).

### Image processing

*MRI*: Brain structural T1-weighted 3D images were acquired in sagittal plane for all subjects on a 3 T Siemens Magnetom scanner using a standard head coil with 1 mm isotropic resolution, TE = 2.96 ms, TR = 2,300 ms, slice thickness = 1 mm, flip angle = 9 deg., FOV = 256 mm, 192 slices per slab. The images were processed following a standard pipeline (Iturria-Medina et al., 2018) including: non-uniformity correction using the N3 algorithm, segmentation into grey matter, white matter and cerebrospinal fluid (CSF) probabilistic maps (SPM12, www.fil.ion.ucl.ac.uk/spm) and standardization of grey matter segmentations to the MNI space (Evans et al., 1994) using the DARTEL tool (Ashburner, 2007). The images were mapped to the Desikian-Killiany-Touriner (DKT) (Klein and Tourville, 2012) atlas for grey matter segmentation. We selected 66 (bilateral) cortical regions that do not present PET off-target binding (Vogel et al., 2020; Sanchez-Rodriguez et al., 2024).

*fMRI*: The resting-state fMRI acquisition parameters were: Siemens Magnetom Prisma, echo planar imaging, 860 time points, TR = 681 ms, TE = 32.0 ms, flip angle = 50 deg., number of slices = 54, slice thickness = 2.5 mm, spatial resolution = 2.5 × 2.5 × 2.5 mm^3^, EPI factor = 88. We applied a minimal processing pipeline (Iturria-Medina et al., 2018) including motion correction, spatial normalization to the MNI space (Evans et al., 1994) and detrending. We then transformed the signals for each voxel to the frequency domain and computed the ratio of the power in the low-frequency range (0.01–0.08 Hz) to that of the entire blood-oxygen-level-dependent (BOLD) frequency range (0–0.25 Hz), i.e., the fractional amplitude of low-frequency fluctuations (fALFF) (Yang et al., 2018; Jia et al., 2019) – a proxy indicator for spontaneous neuronal activity with high sensibility to disease progression (Yang et al., 2018, 2020). The fALFF values were averaged over all voxels belonging to a brain region to yield a single value per region.

*Diffusion Weighted MRI (DW-MRI)*: Additionally, high angular resolution diffusion imaging (HARDI) data was acquired for N = 128 cognitively unimpaired subjects in the Alzheimer’s Disease Neuroimaging Initiative (ADNI) (adni.loni.usc.edu). The authors obtained approval from the ADNI Data Sharing and Publications Committee for data use and publication, see documents http://adni.loni.usc.edu/wp-content/uploads/how_to_apply/ADNI_Data_Use_Agreement.pdf and http://adni.loni.usc.edu/wp-content/uploads/how_to_apply/ADNI_Manuscript_Citations.pdf, respectively (Iturria-Medina et al., 2018). For each diffusion scan, 46 separate images were acquired, with 5 b0 images (no diffusion sensitization) and 41 diffusion-weighted images (b = 1,000 s/mm2). ADNI aligned all raw volumes to the average b0 image, corrected head motion and eddy current distortions. By using a fully automated fiber tractography algorithm (Iturria-Medina et al., 2007) and intravoxel fiber distribution reconstruction (Tournier et al., 2008), we built region-to-region anatomical connection density matrices where each entry, $C_{lk}$ , reflects the fraction of the region’s surface involved in the axonal connection with respect to the total surface of both regions, *l* and *k*. Finally, we obtained a representative anatomical network by averaging all the subject-specific connectivity matrices (Sanchez-Rodriguez et al., 2021). Additional details are available in a previous publication where the data was processed and utilized (Iturria-Medina et al., 2018).

*PET:* Study participants had Aβ (^18^F-NAV4694) and tau (^18^F-MK-6240) PET imaging in a Siemens high-resolution research tomograph. ^18^F-NAV4694 images were acquired approximately 40-70 min after the intravenous bolus injection of the radiotracer and reconstructed using an ordered subset expectation maximization (OSEM) algorithm on a 4D volume with three frames (3 × 600 s)(Therriault et al., 2021). ^18^F-MK-6240 PET scans of 20 min (4 × 300 s) were acquired at 90-110 min post-injection (Pascoal et al., 2020). Images were corrected for attenuation, motion, decay, dead time and random and scattered coincidences and, consequently, spatially normalized to the MNI space using the linear and nonlinear registration parameters obtained for the participants’ structural T1 images. ^18^F-MK-6240 images were meninges-striped in native space before performing any transformations to minimize the influence of meningeal spillover. Standardized Uptake Value Ratios (SUVR) for the DKT grey matter regions were calculated using the cerebellar grey matter as the reference region (Iturria-Medina et al., 2018).

### Estimating Aβ and tau-induced neuronal activity alterations

The subject-specific pathophysiological brain activity was computationally generated through coupled Wilson-Cowan (WC) modules (Wilson and Cowan, 1972; Daffertshofer and van Wijk, 2011; Meijer et al., 2015; Gjorgjieva et al., 2016; van Nifterick et al., 2022) with regional firings mediated by Aβ plaques, tau tangles and the interaction of Aβ and tau (modeled as the product of their across-brain deposition levels) (Sanchez-Rodriguez et al., 2024). Each brain region was dynamically represented through coupled excitatory and inhibitory neural masses (Wilson and Cowan, 1972; Daffertshofer and van Wijk, 2011; Meijer et al., 2015; Gjorgjieva et al., 2016; van Nifterick et al., 2022). Unspecific local inputs and cortico-cortical connections additionally stimulated the excitatory populations. The integration of all inputs was achieved by means of a sigmoidal activation function. In our model, the region-specific excitatory firing thresholds in these sigmoid functions depend on the regions’ accumulation of each pathological factor, an assumption based on findings suggesting neuronal excitability changes due to Aβ and/or tau deposition and the much larger excitatory prevalence in the cortex (Vossel et al., 2017; Busche and Hyman, 2020; Maestú et al., 2021; Targa Dias Anastacio et al., 2022; Tok et al., 2022; van Nifterick et al., 2022). Simplistically, we wrote the effective excitatory firing parameter of participant *j* at brain region *k* as linear fluctuations from the normal baseline value ( $\theta_{0}$ ) due to the considered pathophysiological factors Equation 1:


(1) $$\theta_{j,k}=\theta_{0}+\theta_{j}^{A\beta }\cdot A\beta_{j,k}+\theta_{j}^{Tau}\cdot Tau_{j,k}+\theta_{j}^{A\beta \cdot Tau}\cdot A\beta_{j,k}\cdot Tau_{j,k}$$

Where $A\beta_{j,k}$ and $Tau_{j,k}$ denote the SUVRs normalized to the [0,1] interval –to preserve the dynamical properties of the desired solution–, $\theta_{j}^{A\beta }$ , $\theta_{j}^{Tau}$ and $\theta_{j}^{A\beta \cdot Tau}$ are the brain-wide pathophysiological factor’s influences and each term ( $\theta_{j}^{A\beta }\cdot A\beta_{j,k}$ , $\theta_{j}^{Tau}\cdot Tau_{j,k}$ , $\theta_{j}^{A\beta \cdot Tau}\cdot A\beta_{j,k}\cdot Tau_{j,k}$ ) represents the overall factor’s contribution to neuronal activity in subject *j*’s region *k*.

To estimate these pathophysiological contributions, we simulated BOLD signals. The total action potential arriving to the neuronal populations from other local and external populations (Logothetis et al., 2001) underwent metabolic and hemodynamic transformations following (Sotero and Trujillo-Barreto, 2007, 2008; Valdes-Sosa et al., 2009) to generate the BOLD signal. The full set of differential equations describing these biophysical transformations and operations is provided in Supplementary file. The equations were solved with an explicit Runge–Kutta (4,5) method, ode45, and a timestep of 0.001 s. Then, the parameters $\theta_{j}^{A\beta }$ , $\theta_{j}^{Tau}$ and $\theta_{j}^{A\beta \cdot Tau}$ were obtained via surrogate optimization in MATLAB 2021b (MathWorks, 2021) by maximizing the similarity (i.e., minimizing the correlation distance) between the real and simulated individual BOLD signals’ fALFF indicators (Sanchez-Rodriguez et al., 2024) (see Supplementary file for additional details).

Having obtained the likely individual brain-wide influences due to each of the pathological factors (Aβ, tau and Aβ∙tau), across-brain mechanistic group differences (AD vs. CU) were quantified via the (non-parametric) rank sum test statistics. First, for each subject *j* and brain region, *k*, each pathological factor’s perturbation to neuronal activity in subject *j*’s region *k* was normalized as $\frac{\theta_{j}^{factor}\cdot factor_{j,k}}{\left|\theta_{j,k}-\theta_{0}\right|}$ . Then, the across-regions vectors resulting from the statistical tests (AD vs. CU) quantified the Aβ, tau and Aβ∙tau spatial influences on neuronal activity due to AD.

### Neurotypical gene expression profiles

Microarray mRNA expression data from six neurotypical adult brains was downloaded from the Allen Institute (RRID:SCR_007416) website (http://www.brain-map.org). The data was preprocessed by the Allen Institute to reduce the effects of bias due to batch effects (Hawrylycz et al., 2012; Allen Human Brain Atlas, 2013). For each brain, there were 58,692 probes representing 20,267 unique genes. For genes with multiple probes, Gaussian kernel regression (Gryglewski et al., 2018) was applied to predict the mRNA intensity in each of the 3,702 samples in MNI space (Evans et al., 1994) using leave-one-out cross-validation. The probe with the highest prediction accuracy was chosen as the representative probe for that gene. Gaussian kernel regression using mRNA values of proximal regions also served to predict the gene expression for grey matter voxels without mRNA expression intensity. Thus, the whole-brain gene expression data was obtained for the selected 20,267 probes/genes. Probes/genes described as “uncharacterized,” “similar to hypothetical protein,” “pseudogene” were dropped, leaving 19,469. Finally, we calculated average gene expression values for each region in the brain parcellation (Adewale et al., 2021).

### Molecular associates of the Aβ, tau and Aβ∙tau spatial alterations to neuronal activity

We aimed to determine the genes with whole-brain expressions predicting the Aβ, tau and Aβ∙tau effects on neuronal activity. For each pathological factor, we evaluated monotonic relationships between the corresponding neuronal activity spatial alterations patterns and the regional gene expression values by computing Spearman correlations. We estimated 99% Spearman’s rho confidence intervals with 100,000 bootstrapping resamples and retained the genes which confidence limits did not include zero (significant correlation). The resulting sets of genes were termed Aβ, tau and Aβ∙tau molecular associates, respectively.

### Statistical analyses

We performed functional pathways enrichment analyses on Metascape (Zhou et al., 2019), a web-based portal that integrates various independent biological databases (KEGG Pathway, GO Biological Processes, Reactome Gene Sets, Canonical Pathways, CORUM, WikiPathways, PANTHER Pathway, DisGeNET), using default specifications. Metascape identifies ontology terms that are significantly over-represented in the input gene lists through hypergeometric tests and the Benjamini-Hochberg *p*-value correction algorithm (*q < 0.05*). To avoid redundancy from the reporting of multiple ontologies, Kappa similarities among all pairs of enriched terms are computed. Then, the similarity matrix is hierarchically clustered, and a 0.3 threshold is applied. The most significant (lowest p-value) term within each cluster is chosen to represent the cluster (Zhou et al., 2019). Cell type enrichment was performed with the Expression Weighted Celltype Enrichment toolbox (Skene and Grant, 2016). The probability of enrichment is determined as the percentage of 100,000 random gene lists in a background set with lower average expression in each cell type than in our gene lists. The background gene set is comprised of all genes with orthologs between human and mice and its single-cell transcriptome data were sampled from the mice somatosensory cortex and hippocampus CA1 (Skene and Grant, 2016). Drug repurposing alternatives were investigated on the webserver SigCom LINCS (Evangelista et al., 2022; Xie et al., 2022). This search engine uses a database of ranked gene lists for drug-induced gene expression changes. Similarity and statistical measures (*p*-values, Benjamini-Hochberg corrected, *q < 0.05*) are computed using the Mann–Whitney U test: the average rank of the user-provided gene set in each chemical perturbation’s gene list is compared to the average rank of a randomly selected gene set (Evangelista et al., 2022; Xie et al., 2022).

## Results

### Brain-wide neuronal dysfunction in AD associate with spatially distinctive molecular signatures

**Figure 1:**
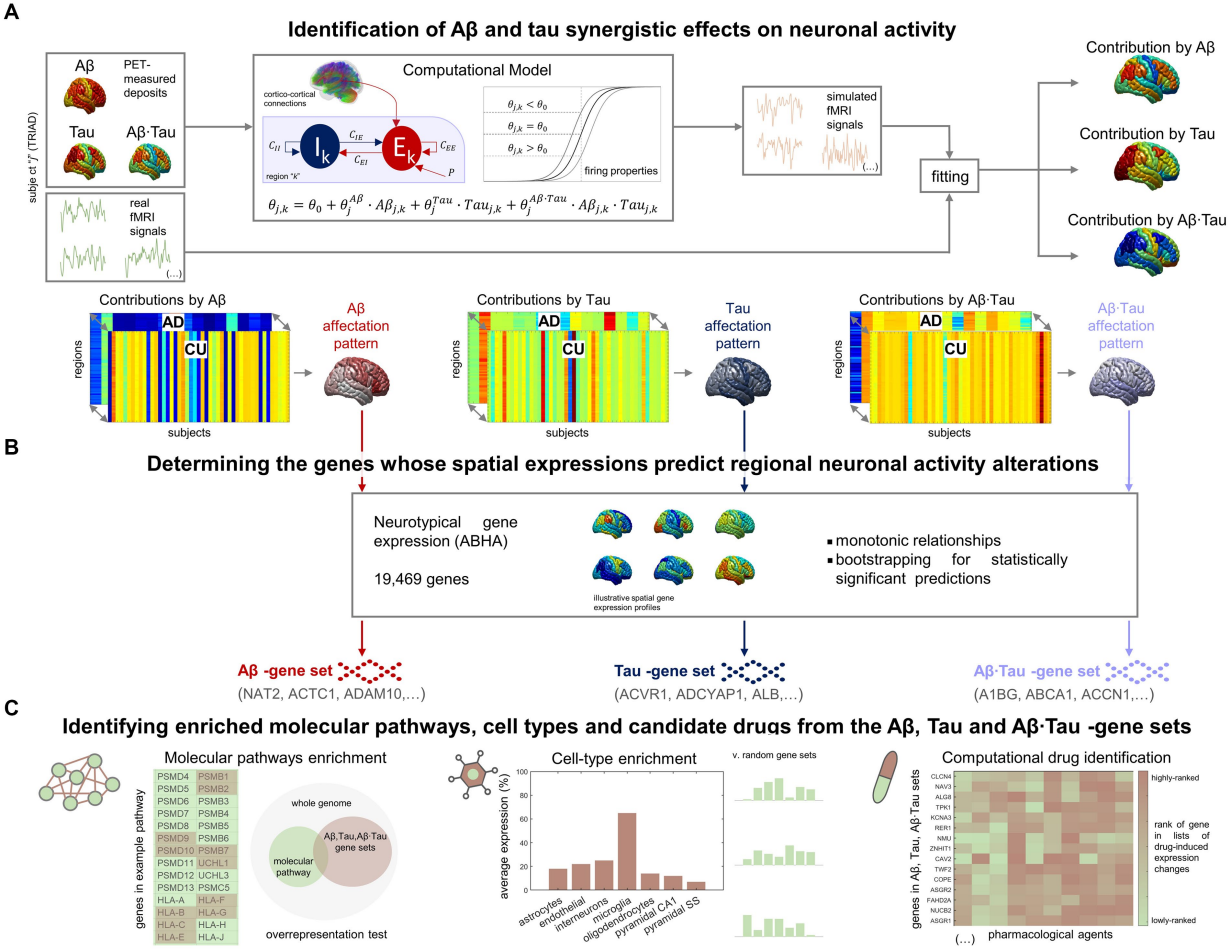
Approach for determining molecular mechanisms spatially associated with Aβ and tau-induced neuronal dysfunction in AD. **(A)** For each participant in the TRIAD cohort, neuronal excitability within a brain region depends on the combined Aβ and tau accumulations. The generated excitatory and inhibitory activities are transformed into fMRI signals. The most-likely *in-vivo* subject-specific Aβ and tau effects are obtained through maximizing the similarity of the generated fMRI signals with the participant’s real resting-state fMRI across all regions. Statistical comparison of the obtained regional Aβ, tau and Aβ∙tau contributions to pathophysiological neuronal activity between the AD and the Aβ- and tau-negative CU groups yields spatial alterations patterns by each of these disease factors in AD (the higher the statistic, the more different the groups are). **(B)** Next, we investigated spatial correlations with neurotypical whole-brain transcriptomics (99% bootstrap confidence intervals) and obtained the genes which expressions predict the regional neuronal activity effects by Aβ, tau and Aβ∙tau. **(C)** The sets of Aβ, tau and Aβ∙tau associates serve to identify enriched biological processes (molecular pathways from multiple gene ontologies that are overrepresented) (Zhou et al., 2019), brain cell-types (the Aβ, tau and Aβ∙tau gene sets having higher expression for a particular cell type than what is expected by chance) (Skene and Grant, 2016) and prospective pharmacological agents to halt or reduce AD-affected processes (by comparing the gene sets to databases of drug-induced gene expression changes) (Evangelista et al., 2022).

In our investigation into the molecular processes predicting pathophysiological alterations in the AD brain, we divided our research into two main components. First, we quantified *in-vivo* AD-characteristic neuronal activity alterations using data from the TRIAD database (Figure 1A). We employed personalized computational models (Wilson and Cowan, 1972; Sotero and Trujillo-Barreto, 2007; Valdes-Sosa et al., 2009) informed by the participants’ neuroimaging (fMRI, Aβ- and tau-PET) (Sanchez-Rodriguez et al., 2024). For each AD and CU subject (Supplementary Table S1), we assumed that neuronal excitability across the brain’s gray matter regions (DKT parcellation (Klein and Tourville, 2012)) was potentially influenced by the local Aβ and tau accumulations. Functional alterations by Aβ and tau spatiotemporally transmit through intra-regional and cortico-cortical connections derived from diffusion MRI (Iturria-Medina et al., 2018). Through individualized modeling, we generated in-silico pathophysiological excitatory and inhibitory activities (Wilson and Cowan, 1972) which were transformed into blood-oxygen-level-dependent (BOLD) signals (Sotero and Trujillo-Barreto, 2007; Valdes-Sosa et al., 2009). Subject-specific contributions by each factor Aβ, tau and Aβ∙tau (their synergistic interaction) were derived by fitting the in-silico BOLD signals to the subject’s real regional resting-state fMRI content within the physiologically-relevant neuronal activity range (0.01–0.08 Hz) (Yang et al., 2018; Sanchez-Rodriguez et al., 2024). This approach enabled us to identify distinctive spatial Aβ, tau, and Aβ∙tau neuronal activity alteration patterns via statistical evaluation of the neuronal excitability perturbations in the AD vs. CU groups. Second, we investigated statistical relationships with spatial gene expression profiles in the human transcriptome (Figure 1B). Average expression values of all genes in the Allen Human Brain Atlas (AHBA) were calculated for each region in the parcellation, using post-mortem data from six adult neurotypical brains (Hawrylycz et al., 2012; Allen Human Brain Atlas, 2013; Adewale et al., 2021). By computing 99% bootstrap confidence intervals for the brain-wide correlations between the Aβ, tau and Aβ∙tau spatial patterns and the expression of each gene, we identified the genes from the post-mortem human transcriptome whose spatial expressions predict the *in-vivo* neuronal activity effects that are induced by each pathophysiological component (Aβ, tau and Aβ∙tau).

We found 756, 650 and 1987 genes, respectively, in the Aβ, tau and Aβ∙tau-associated gene sets. The detected genes (Supplementary Table S2) include several previously associated with AD risk (Calabrò et al., 2021). Notably, *SNCA* (synuclein Α) is essential for presynaptic signaling and membrane transport and participates in NFT formation and Aβ deposits (Calabrò et al., 2021). The protein encoded by the gene *CLU* (clusterin) inhibits Aβ fibrils formation (Calabrò et al., 2021). Gene *ADAM10* (α disintegrin and metalloproteinase domain-containing protein 10) plays a critical role in cleavage of the amyloid precursor protein (APP) (Calabrò et al., 2021). Finally, the microglial activation modulator *CD33* (Sialic Acid-Binding Ig-Like Lectin 3) is one of the top-ranked genetic factors identified in AD genome-wide association studies (Zhao, 2019). As reported in the next subsections, the Aβ, tau and Aβ∙tau molecular associates of AD pathogenesis were further investigated in terms of overrepresented biological mechanisms, cellular types associated with brain-wide functional affectations and pharmacological agents with potential therapeutic benefit (Figure 1C).

### Immune and cell communication patterns relate to AD pathology-induced neuronal dysfunction

**Figure 2:**
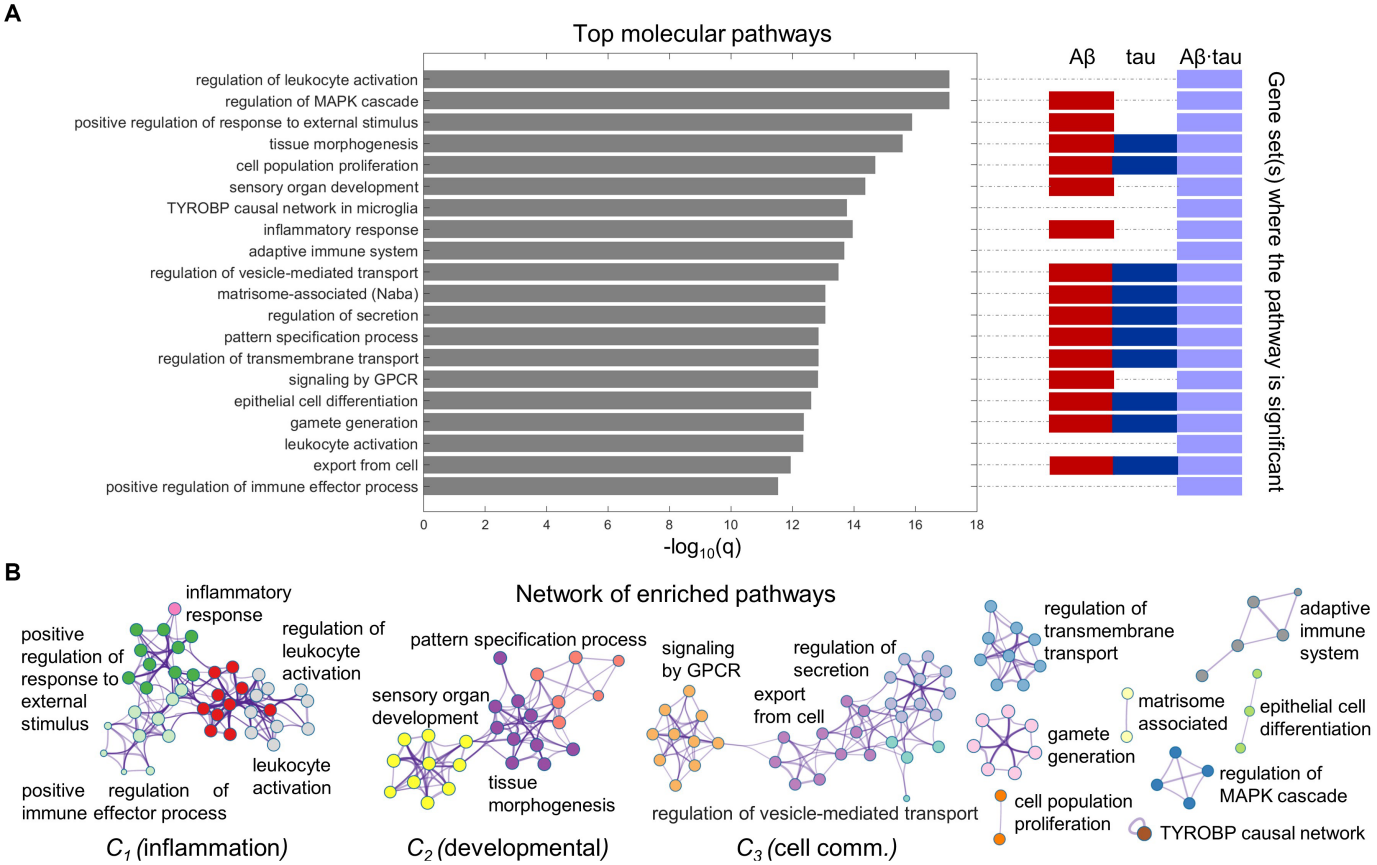
Neuroinflammation pathways emerge as major processes associated with Aβ-tau interactions. **(A)** Top 20 pathways clusters from multiple gene ontologies that are enriched in the combined Aβ-, tau- and Aβ∙tau-associated gene sets (hypergeometric tests, *q* < 0.05, Benjamini-Hochberg corrected). The representative biological processes (term with the lowest *p*-value within a cluster) are used as labels. Additionally, the specific pathological factors for which the pathways are statistically significant have been indicated next to the bar graph. **(B)** Intra- and inter-cluster similarities among the obtained molecular processes. Each node represents an enriched pathway. The network is colored by the cluster labels, which are written next to each cluster. Note that major clusters include neuroinflammation and immune system processes (C_1_), developmental pathways (C_2_) and cell communication mechanisms (C_3_).

We proceeded to functionally interrogate the three neuronal dysfunction gene sets (specific to Aβ, tau and Aβ∙tau effects) with ontology terms from various sources in Metascape (Zhou et al., 2019), detecting the associated molecular pathways (Supplementary Table S3). Figure 2 summarizes the identified molecular mechanisms associated with the causal combined roles of Aβ and tau pathologies on AD’s neuronal activity alterations. The top 20 enriched functional clusters that were detected, together with the gene lists where the pathways were found statistically significant (hypergeometric tests, FDR-corrected, *q* < 0.05) are shown in Figure 2A. In addition, all the Aβ + tau → neuronal-activity genes that are consistently involved (95% percentile) within the top statistically significant biological pathways are reported in Supplementary Table S4. Top genetic mediators, e.g., *RIPK2*, *SYK*, *ANXA1* and *SNCA,* have documented roles in the formation/response to Aβ and tau deposits and in microglial activation (Natarajan et al., 2013; Twohig and Nielsen, 2019; You et al., 2021; Ennerfelt et al., 2022).

Pathway-pathway similarities based on genetic overlap (Zhou et al., 2019) are visualized in the network space (Figure 2B). Notably, we observed strong clustering of various neuroinflammation and immune system pathways. For instance, *inflammatory response* connects with the *positive regulation of immune effector process*, *positive regulation of response to external stimulus* and *leukocyte activation*. This unsupervised result is aligned with the fact that persistent chronic inflammation, due to genetic and lifestyle factors, plays a key role at the onset and later progression of neurodegeneration (Newcombe et al., 2018; Calabrò et al., 2021). It has been hypothesized that Aβ and tau accumulation can both trigger and be triggered by disbalanced inflammatory signals (Newcombe et al., 2018). Another identified functional cluster includes critical developmental processes (*sensory organ development*, *tissue morphogenesis*, *pattern specification process*). Cell communication/transport mechanisms, fundamental to proper synaptic function and implicated in AD pathogenesis according to several reports (Gadhave et al., 2021) were also found among the top enriched molecular processes in a major cluster (*regulation of secretion, regulation of vesicle-mediated transport, export from cell, signaling by GPCR*; Figure 2B).

Additionally, we examined biological processes separately related to the Aβ, tau and Aβ∙tau gene sets (Supplementary Table S3). Immune system pathways were once again overrepresented in the Aβ∙tau set, while developmental and synaptic processes were enriched for Aβ’s molecular associates. Notably, some pathways that ranked lower in the integrative analysis in Figure 2, had strong associations with the tau-associated gene list (with less elements than the Aβ and Aβ∙tau molecular signatures). Amongst the enriched terms, several supposedly tau-related processes (Mandelkow and Mandelkow, 2011; Bennett et al., 2018) including *cortical cytoskeleton organization*, *regulation of actin filament organization*, *blood vessel development* and *post-translational protein phosphorylation* appeared.

Next, we explored molecular overlap with other diseases according to the genes predicting the spatial neuronal activity combined Aβ and tau effects. We determined which disease pathways, curated in DisGeNET (Piñero et al., 2017; Zhou et al., 2019), were enriched in our gene sets (Supplementary Figure S1). Notably, the obtained enriched terms include several infection and immunological conditions (e.g., immunosuppression, Behcet syndrome and lupus), certain cancers, and eye diseases, for the three considered sets of molecular associates. Likewise, we retrieved characteristic AD phenotypical symptoms (Ghiso and Frangione, 2002; Bennett et al., 2013) such as *memory impairment* (enriched in both Aβ and tau signatures) and *amyloidosis* (Aβ). Together, these results substantiate the idea that our approach unifying whole-brain transcriptomics, functional neuroimaging, and personalized computer-simulated neuronal activity can reproduce and identify major disease mechanisms and manifestations.

### Microglia, pyramidal cells and interneurons at the core of AD dysfunction

**Figure 3:**
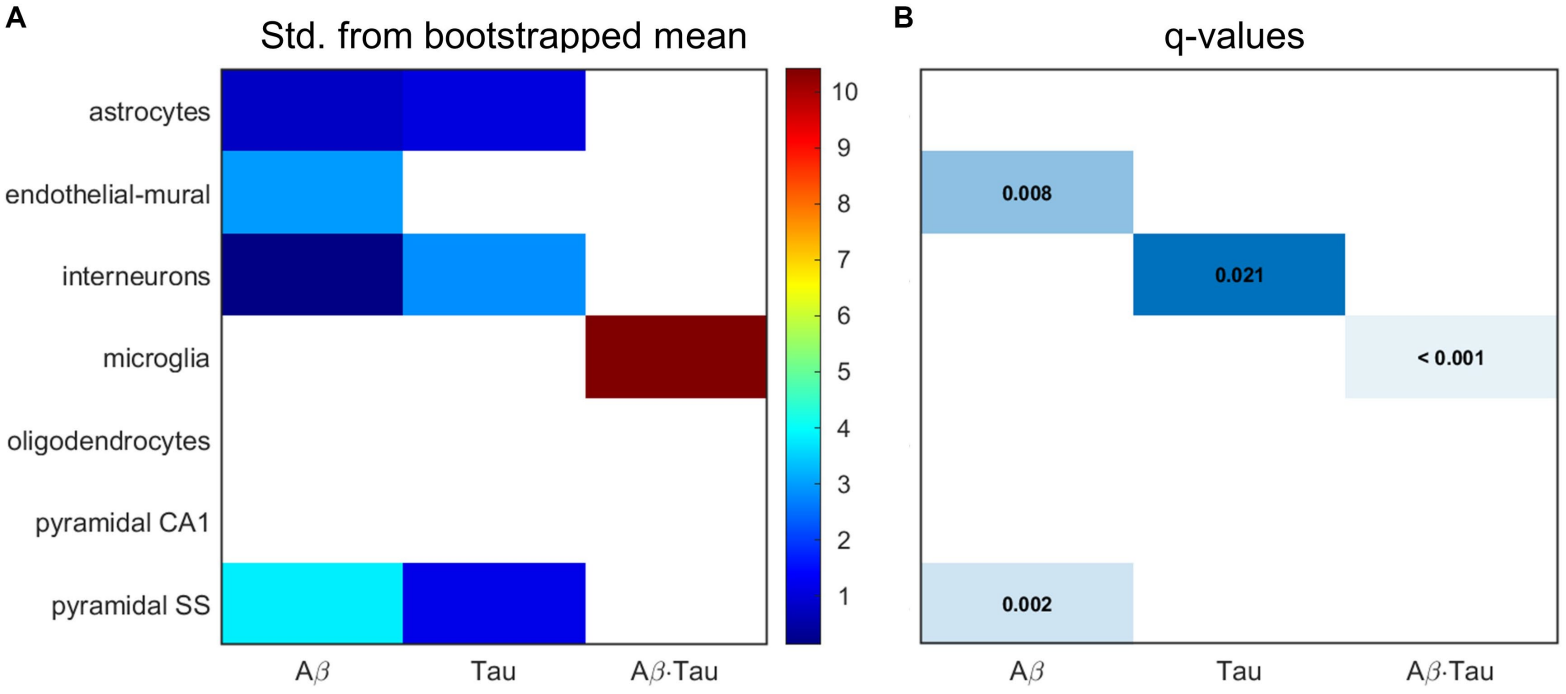
Transcriptomic associates of neuronal activity alterations induced by Aβ and tau converge to neuro-vascular cellular compartments. Results of bootstrapping tests to evaluate the probability of the Aβ-, tau- and Aβ∙tau-associated gene sets having higher expression for a particular brain cell type than what is expected by chance. **(A)** Number of standard deviations from the bootstrapped mean for every gene set and cell type. Non-white boxes indicate that the given molecular associates’ expression in the specific cell type is, on average, higher than that of the bootstrapped sets. **(B)** Statistically significant enrichment (*q* < 0.05, Benjamini-Hochberg corrected).

Next, we hypothesized that the gene sets associated with each of the pathophysiological neuronal activity patterns would be particularly enriched in distinct cell types. We performed a bootstrapping-based cell type enrichment analysis on the Expression Weighted Celltype Enrichment toolbox (Skene and Grant, 2016) and determined the statistical likelihood of brain cell types being enriched compared to the background gene set (Figure 3).

Microglia presented strong enrichment for the Aβ∙tau signature gene set (*q* < 0.001 and *δ* = 10.425, number of standard deviations from the bootstrapped mean). To our knowledge, neuronal dysfunction due to Aβ and tau interactions have never been studied in the context of genetic cell enrichment although analyses of the disease’s polygenic post-mortem expression have also found damage to microglia (Galatro et al., 2017; Newcombe et al., 2018). Additionally, we observed evidence supporting pyramidal cells (*q* = 0.002 and *δ* = 3.804) and endothelial-mural cells (*q* = 0.008 and *δ* = 2.950) as the most enriched cell types amongst the Aβ molecular associates. Pyramidal neurons, the most abundant neural cells in the cortex, are known to be a preferential target for Aβ toxic deposits (Maestú et al., 2021). Previous studies (Koizumi et al., 2016) also showed impairment to cerebral blood vessels –composed of endothelial and mural cells– by extracellular buildup of Aβ, while vascular dysfunction may promote Aβ accumulation in a detrimental feedback loop. On the other hand, the tau susceptibility genes had significant interneuron expression according to the bootstrapping analysis (*q* = 0.021 and *δ* = 2.834). Phosphorylated tau accumulates early in hippocampal interneurons of AD patients, impairing adult neurogenesis and circuital function (Xu et al., 2020; Zheng et al., 2020). Overall, these results support that different cellular vulnerability patterns relate to spatial neuronal activity alterations induced by Aβ, tau and Aβ∙tau pathophysiological mechanisms.

### Immunologic compounds may halt or reduce AD neuronal dysfunction

Finally, we examined whether existing pharmacological agents could be utilized to target AD’s identified Aβ and tau effects on neuronal activity. We compared the identified Aβ-, tau-, and Aβ∙tau-associated gene sets to databases of drug-induced gene expression changes using SigCom LINCS (Evangelista et al., 2022) and detected chemical compounds that maximally upregulate or downregulate these gene lists. Table 1 presents the top statistically significant (*q* < 0.05) candidate drugs that have also been FDA-approved (for a full list of all identified compounds, see Supplementary Table S5). Drug indications (accessed through https://pubchem.ncbi.nlm.nih.gov/ on May 10th, 2023) and blood–brain barrier (BBB) permeabilities (Meng et al., 2021) of the top prospective repurposed medications are also provided (see also Supplementary Figure S2). In separate analyses, we additionally queried drug-molecular targets interactions of the independent Aβ, tau and Aβ∙tau-associated gene sets (Supplementary Table S5).

**Table 1:** Top identified drug repurposing candidates to target adverse Aβ- and tau-induced neuronal-activity effects.

Pharmacological agent	Drug use indications	Regulatory response	*Z*-scores	BBB permeability	Isolated set targets
*Cancer*					
Doxorubicin			7.326	BBB+	Aβ∙tau
Daunorubicin			7.302	BBB+	Aβ∙tau
Ponatinib			7.134	BBB-	–
Vorinostat			6.942	n.a.	Aβ, tau
Epirubicin			6.985	BBB-	Aβ∙tau
Cytarabine			6.445	BBB+	–
Dasatinib			6.433	BBB+	–
Dinaciclib		Up ↑	6.403	n.a.	–
Afatinib		Down ↓	-7.410	n.a.	–
Azacitidine			-7.093	BBB-	Aβ∙tau
Thioguanine			-6.883	BBB-	–
Duvelisib			-6.809	n.a.	–
Bosutinib			-6.745	BBB-	–
Gefitinib			-6.472	BBB+	Aβ∙tau
Rucaparib			-6.268	n.a.	Aβ∙tau
Selumetinib			-6.248	n.a.	Aβ, Aβ∙tau
*Immune*					
Triptolide			7.731	n.a.	tau, Aβ∙tau
Auranofin			6.475	n.a.	Aβ∙tau
Mycophenolic acid		Up ↑	6.427	BBB-	Aβ∙tau
Diclofenac		Down ↓	-6.634	BBB+	–
Niclosamide			-6.596	BBB+	Aβ∙tau
Ritonavir			-6.347	BBB-	–
Isotretinoin			-6.283	BBB+	–
Famciclovir			-6.265	BBB+	Aβ∙tau
Filgotinib			-6.233	n.a.	–
*Eye*					
Levocabastine			7.049	BBB+	Aβ∙tau
Varenicline		Up ↑	6.899	BBB+	Aβ∙tau
Nicergoline		Down ↓	-6.723	BBB+	Aβ∙tau
Docosahexaenoic acid			-6.423	n.a.	–
*Cardiac*					
Verapamil		Up ↑	6.419	BBB+	Aβ∙tau
Apixaban		Down ↓	-6.370	BBB-	–
*Sclerosis*					
Mitoxantrone			8.425	BBB-	Aβ∙tau
Riluzole		Up ↑	6.428	BBB+	Aβ∙tau
*Others*					
Amisulpride		Down ↓	-6.585	BBB+	Aβ∙tau
Estradiol			-6.572	BBB-	–
Ramelteon			-6.326	BBB+	–

Reported are existing drugs which molecular interactions would induce gene expression changes in the set of all Aβ-, tau- and Aβ∙tau-associated genes (Mann–Whitney *U* test, *q* < 0.05, Benjamini-Hochberg corrected). The predicted chemical compounds have been organized in major groups according to their drug use indications. All medications are FDA-cleared for either treatment of cancer, various immune system/infection/inflammatory processes (“immune”), eye diseases, cardiovascular conditions, multiple or amyotrophic lateral sclerosis or “other” disorders. The groups are further divided by whether the candidate drug up- or down-regulates the genes linked to the neuronal activity alterations by AD. Additionally, blood–brain barrier permeability, is specified (BBB+: permeable to the blood–brain barrier; BBB-: not permeable to the blood–brain barrier; n.a.: information not available). Drugs that could also target the separate Aβ, tau or Aβ∙tau molecular associates are identified in the last column, e.g., the chemical selumetinib may be used to modify Aβ- and Aβ∙tau- associated gene sets.

The identified chemical compounds with the capacity to target neuronal-activity dysfunction due to Aβ and tau are, mostly, drugs already used for the treatment of immune system-related disorders and cancer. Among the top immunological drug candidates, the immunosuppressant medication mycophenolic acid, indicated for prophylaxis of organ rejection, has been reported to attenuate neuronal cell death (Ebrahimi et al., 2012); diclofenac could potentially associate with reduced AD risk and slower cognitive deterioration (Rivers-Auty et al., 2020), while antiherpetic medication as famciclovir may also prevent AD incidence (Calabrò et al., 2021; Linard et al., 2022). Likewise, anti-inflammatory multiple sclerosis medication has shown promise in AD mouse models for reversing all Aβ, tau and microglia pathologies, and synaptic and cognitive dysfunction (Dionisio-Santos et al., 2021; Leßmann et al., 2023). However, it is worth noticing that drugs with anti-inflammatories properties have not slowed cognitive and/or functional decline in clinical trials (Howard et al., 2020; Melchiorri et al., 2023). One possible explanation is that the thus-far tested agents interfere with microglia’s supportive function instead of modulating its detrimental chronic activation effects (Shen et al., 2018; Howard et al., 2020; Rivers-Auty et al., 2020; Melchiorri et al., 2023). At least 18 investigational drugs targeting neuroinflammation currently undergo clinical assessment, including phase III trials (Reading et al., 2021; Melchiorri et al., 2023).

Common indications among the identified cancer medications include leukemia, lymphoma and breast cancer. In clinical research, prospective disease-modifying AD drugs commonly target cancer pathways (Morgan et al., 2022). Other computational drug repurposing studies have similarly suggested the potential benefits of anti-cancer drugs. For example, a multi-omics study identified interactions of afatinib, dasatinib, gefitinib and ponatinib with AD-affected genes (e.g., *APP*, *SNCA*) (Advani and Kumar, 2021). Within the remaining identified prospective candidates, cardiovascular drugs may lower the incidence of dementia –apixaban (Bezabhe et al., 2022)– and delay progression in a mouse model of AD –verapamil (Ahmed et al., 2021). Additionally, docosahexaenoic acid (omega-3) supplementation has been linked to reduced AD risks (Quinn et al., 2010; Arellanes et al., 2020). Randomized trials finding interactions with *APOE4* suggest that such AD carriers could also potentially present favorable imaging and cognitive outcomes with high dose docosahexaenoic acid supplementation treatments (Arellanes et al., 2020). On the other hand, retinopathy, glaucoma and age-related macular degeneration are deemed prominent signs of AD pathology (Mirzaei et al., 2020), functionally sharing affected molecular pathways (Supplementary Figure S1), which explains the appearance of visual impairments medication among the top prospective drugs. These data-driven results suggest therapeutic alternatives to be tested in randomized controlled trials (RCTs) for the treatment and prevention of AD, bypassing the early stages of drug design for compounds with known pharmacokinetic/pharmacodynamic properties.

## Discussion

AD research has proposed a myriad of interacting mechanisms with potential central contributions by Aβ and tau (Iturria-Medina et al., 2018; Newcombe et al., 2018; Maestú et al., 2021; Therriault et al., 2022; Sanchez-Rodriguez et al., 2024). However, molecular and cellular mediators of their impact on neuronal activity remain elusive and little is known about disease mechanisms in the living human AD brain. Using an integrative computational approach informed by *in-vivo* neuroimaging of AD patients and cognitively unimpaired (CU) subjects negative for both Aβ and tau, along with *ex-vivo* neurotypical whole-brain transcriptomics, we investigated neuronal activity alterations induced by both Aβ and tau pathologies in AD and mapped their spatial overlap with neurotypical gene expression. The study’s main contributions are as follows: (1) identifying molecular and cellular patterns that spatially overlap with dysregulations in neuronal activity caused by Aβ and tau, (2) exploring the combined impact of Aβ and tau on various biological processes, rather than focusing solely on isolated disease mechanisms, and (3) taking a bottom-up translational approach, starting from the discovery of spatial disease molecular signatures and progressing to potential disease-modifying interventions targeting functional-neuropathological pathways.

Several of the identified spatial molecular correlates from the human brain transcriptome have been linked to AD in the past (Calabrò et al., 2021). For example, the *SNCA* gene translates into the presynaptic protein α-synuclein, which presents high concentration in the cerebrospinal fluid of mild cognitive impairment and AD patients and forms deposits that have been found in the majority of autopsied AD brains (Twohig and Nielsen, 2019). Gene *RIPK2* is a mediator of mitochondrial dysfunction in oligodendrocytes and demyelination (Natarajan et al., 2013), *SYK* coordinates neuroprotective microglial response to Aβ pathology (Ennerfelt et al., 2022) and *ANXA1* plays an important role in controlling neuronal damage by immune responses (You et al., 2021). All these molecules appear in most of the top overrepresented biological processes (Supplementary Table S4) and are central to the overall pathophysiological-molecular signature.

The pathways enriched within AD’s Aβ + tau → neuronal-activity molecular signature likely represent key biological processes associated with brain dysfunction in AD. Our analyses utilized comprehensive and robust resources (Skene and Grant, 2016; Zhou et al., 2019; Evangelista et al., 2022) –e.g., Metascape integrates major current biological databases including KEGG Pathway, GO Biological Processes, WikiPathways and PANTHER Pathway. Our findings are consistent with the existing literature. For instance, we detected leukocyte activation (and its regulation) among the top molecular pathways, which aligns with genetic associations linking specific types of blood leukocytes to the risk of Alzheimer’s disease (Luo et al., 2022). Other immune-related biological processes such as positive regulation of response to external stimuli and positive regulation of cytokine production (as depicted in Figure 2 and Supplementary Table S3) have recently been identified through analyzing differentially expressed genes between Alzheimer’s disease and control groups (Zhao et al., 2022). Moreover, we found sensory organ development pathways related to the eye and retina, considered early markers of the disease (Mirzaei et al., 2020; Koronyo et al., 2023). Retinal changes, including an overabundance of Aβ42, correlate with Braak cortical tau involvement and cognitive decline in AD patients. Among the identified cell communication/transport mechanisms, the regulation of protein secretion and transport may be crucial for controlling tau and Aβ levels (Annadurai et al., 2021; Calabrò et al., 2021). Additionally, G protein-coupled receptors (GPCRs) are affected by Aβ peptides, leading to synaptic loss and impaired neurotransmission in AD (Gadhave et al., 2021). Memory impairment and other hallmark signs of AD, including amyloidosis and phosphorylation (Ghiso and Frangione, 2002; Mandelkow and Mandelkow, 2011; Bennett et al., 2013), were also overrepresented in Aβ and tau’s molecular associates of pathophysiological neuronal activity. We have confirmed existing hypotheses from cell culture, animal and post-mortem research regarding AD as a virtually generalized condition –see for example the recent review by Calabrò et al. (2021) and text-mining of the AD literature by Morgan et al. (2022).

Nevertheless, our integrative estimations indicate that neuronal activity alterations by Aβ, tau and their synergistic interaction are consistently related to inflammation processes, further demonstrating their fundamental role in AD’s *in-vivo* human pathophysiology. Peripheral immune cells, through disruptions to the central nervous system borders (e.g., BBB leakage) have potential major contributions to AD pathogenesis (Jorfi et al., 2023). In addition, pro-inflammatory microglial activation/neuroinflammation may trigger (or interact in) different pathological processes (Shen et al., 2018; Kwon and Koh, 2020; Calabrò et al., 2021; Jorfi et al., 2023). We found that the spatial molecular associates of the interaction between Aβ and tau pathologies were more enriched for microglial expression than expected by chance. Previous studies have suggested that prolonged, uncontrolled immune responses cascade to modify physiological properties and the neuronal activity balance through interactions with Aβ and tau (Newcombe et al., 2018; Shen et al., 2018; Kwon and Koh, 2020; Calabrò et al., 2021). Our findings indicate that neuroinflammation also interplays with Aβ and tau synergistic effects, which seems to be a key factor in AD’s pathophysiology (Busche and Hyman, 2020; Sanchez-Rodriguez et al., 2024). The identification of a major cluster of immunological pathways within AD’s neuronal activity molecular signatures warrants further investigation. In our previous work (Sanchez-Rodriguez et al., 2024), we sought to decode possible neuroinflammatory influences (interacting with Aβ and tau effects) to neuronal activity through personalized computational models. However, only slight significant differences in the translocator protein microglial activation -PET data existed between AD and CU subjects, underscoring broadly discussed limitations of PET tracers being unspecific to inflammatory variants (Shen et al., 2018; Nutma et al., 2023).

Further improvements and clinical validation are necessary for implementing treatment strategies suggested by computational modeling of neuropathological mechanisms (Iturria-Medina et al., 2018; Maestú et al., 2021), as this study presents several limitations. The TRIAD dataset utilized in the study was collected at a specialist memory clinic that receives relatively young dementia patients. This highly specialized setting may pose a limitation in terms of generalizability, although subjects diagnosed as “early-onset” and/or “familial” AD were excluded from the current analysis. Additionally, the percentage of female subjects within the CU (AD) group was slightly higher (lower) than AD’s prevalence among women, i.e., nearly two-thirds of the total number of cases (World Alzheimer Report, 2022). Likewise, the sub-cohort was not balanced and small (47 CU vs. 16 AD individuals). Several factors contributed to these disproportions including the availability of volunteers and whether the necessary imaging modalities had been collected at the time of sample curation (i.e., we selected all existing AD subjects and contrasted them to CU participants who were negative for both Aβ and tau, Supplementary Table S1). More advanced implementations of our approach would also consider disease heterogeneity, detecting sub-trajectories (Iturria-Medina et al., 2020, 2021) over the AD spectrum and obtaining molecular affectation signatures for each of those phenotypes, which was not statistically viable in the present study due to the relatively small sample size. Regarding the biophysical model for neuronal activity alterations due to AD’s pathology, we considered perturbations to pyramidal neurons only. Albeit a sound approximation given the pyramidal preponderance in the cortex (Maestú et al., 2021) –and with local connections propagating alterations to inhibitory populations as well (Wilson and Cowan, 1972)– this assumption could be relaxed by considering an inhibitory influence model and re-estimating the relevant pathophysiological parameters. By doing so, we may test hypotheses for inhibitory circuit impairment in AD (Zheng et al., 2020; Maestú et al., 2021; Targa Dias Anastacio et al., 2022). Our observations also necessitate further validation to fully comprehend the causal synergistic effects of Aβ and tau across different brain areas. A main issue is that we combined neuronal activity indicators derived from *in-vivo* neuroimaging assessments in the TRIAD cohort with the neurotypical AHBA gene expressions profiles. This fusion is necessitated due to the current absence of brain-wide genomic AD measurements (Gabitto et al., 2023; Ng et al., 2023). While this methodology is common in the literature (Mullins and Kapogiannis, 2022; Ye et al., 2022; Tang et al., 2024), it overlooks individual variations and disease-specific transcriptomic dysregulations. In future research, we intend to overcome these limitations by extending our analyses to large-scale cohorts that include both ante-mortem neuroimaging and post-mortem gene expression data. As tissue coverage expands in post-mortem AD brains (Gabitto et al., 2023; Ng et al., 2023), we aim to utilize these resources for more comprehensive explorations of overrepresented signaling pathways and cell types.

Importantly, we focused the scope of this investigation into AD neuronal dysfunction by Aβ and tau only. The identified subject-specific Aβ and tau neuronal activity alterations should be interpreted as their causal pathophysiological effects disregarding other possible contributors (e.g., vascular, immune). It is known that additional factors as glial cell activity affects neuronal firing, even in healthy states (Targa Dias Anastacio et al., 2022). In effect, our personalized models are readily modifiable (Sanchez-Rodriguez et al., 2024) to consider other pathological factors, provided that the corresponding brain maps are available. Advanced causal computational models unifying neuroimaging and omics exist (Adewale et al., 2021; Iturria-Medina et al., 2021, 2022; Khan et al., 2022; Lenglos et al., 2022), although they have yet to tackle the generation of (pathophysiological) neuronal activity. In future work, we intend to expand the high-dimensionality, multimodal approaches compiled within the in-house open-access NeuroPM-box software (Iturria-Medina et al., 2021) with quantification tools for unveiling molecular mechanics of pathological influences on neuronal activity. It is imperative to improve our understanding of the causal role that all possible neuropathological players have as this will also allow their early modification through healthy lifestyle choices and clinical monitoring, boosting disease prevention (Silva et al., 2019; World Alzheimer Report, 2022).

The disease-oriented computational drug repurposing strategy that we present constitutes an accelerated alternative to costly drug development for AD, as preliminary safety and bioavailability criteria are already established for existing drugs (Corbett et al., 2012; Mullen et al., 2016; Petralia et al., 2022). In 2021, approximately 40% of Alzheimer’s trials registered on ClinicalTrials.gov used repurposed medication (Cummings et al., 2021). Here, we have delved into the molecular mechanisms linked to the synergistic, across-brain pathological impact on *in-vivo* neuronal activity and searched for disease-modifying agents in the Library of Integrated Network-Based Cellular Signatures (LINCS) (thousands of perturbagens characterized at a variety of time points, doses, and cell lines) (Evangelista et al., 2022; Xie et al., 2022). Potential pharmaceutical interventions were statistically identified and ranked based on the similarity between their documented mechanisms of action and the gene sets of neuronal dysfunctions by AD. Previous studies (Ebrahimi et al., 2012; Arellanes et al., 2020; Rivers-Auty et al., 2020; Advani and Kumar, 2021; Ahmed et al., 2021; Dionisio-Santos et al., 2021; Bezabhe et al., 2022; Linard et al., 2022; Leßmann et al., 2023) have assessed the usefulness of several of our discovered candidate pharmacological agents targeting affected AD pathways (Table 1). Most of these compounds are blood cancers and rheumatoid arthritis drugs with anti-inflammatory properties, which were also pinpointed as viable candidates to halt or reduce AD affectations in a whole-brain transcriptomics machine learning approach (from a pool of 80 FDA-approved and clinically tested drugs) (Rodriguez et al., 2021). Converging evidence indicates that cancer treatment may be related to a decreased risk of AD due to a pathophysiological overlap between both diseases, albeit a worsened cognition being in some studies linked to oncology drugs (Plun-Favreau et al., 2010; Frain et al., 2017; Chen et al., 2021). The FDA-approved compound dasatinib, for the treatment of chronic myeloid leukemia, and one of the top up-regulators identified in our search, has reduced tau pathology in mice (Roberts et al., 2021) and is the subject of an ongoing clinical study evaluating its feasibility and efficacy modulating AD’s progression in combination with the naturally derived anti-inflammatory quercetin (Advani and Kumar, 2021; Gonzales et al., 2022). Although the emphasis of our discussion was on repurposed drugs, other unapproved small molecules (Supplementary Table S5) could also modify the detected AD targets. The identified chemical compounds could be considered for clinical investigation in AD based on several factors, including their specific genetic targets (e.g., Aβ∙tau molecular associates), desired therapeutic response, blood–brain barrier permeability, potential adverse effects, etc. Relevant information is available in our results (see Table 1 and Supplementary Table S5) and the consulted databases (Meng et al., 2021; Evangelista et al., 2022; Xie et al., 2022; https://pubchem.ncbi.nlm.nih.gov/). Mechanistic characterizations, such as those provided in our study, play a crucial role in facilitating the discovery and development of therapeutics, which could potentially increase the effectiveness of randomized controlled trials (Corbett et al., 2012; Mullen et al., 2016; Iturria-Medina et al., 2018; Jack et al., 2018; Cummings et al., 2021; Rodriguez et al., 2021; Petralia et al., 2022). In the future, clinicians may tailor treatment approaches to target the patient’s unique pathological biomarkers using combination therapies and pleiotropic drugs, aiming for universal and more effective disease-modifying outcomes.

## Data availability statement

The datasets presented in this article are not readily available due to their containing information that could compromise the privacy of research participants. PET and resting-state fMRI data utilized in the study are available by submitting a data share request via https://triad.tnl-mcgill.com/contact-us/. All the data collected under the TRIAD cohort is governed by the policies set by the Research Ethics Board Office of the McGill University, Montreal and the Douglas Research Center, Verdun. Microarray mRNA expression data from six neurotypical adult brains is available from the Allen Institute (http://www.brain-map.org). The identified gene sets and consequent analyses are provided in the Supplementary file. The code utilized in this article for the neuronal activity simulations and quantification of the pathological effects can be accessed at the Neuroinformatics for Personalized Medicine lab’s website (NeuroPM, https://www.neuropm-lab.com/publication-codes.html) and is freely available and documented on the Zenodo repository. Supplementary file contains the algorithm’s overview. Requests to access the datasets should be directed to https://triad.tnl-mcgill.com/contact-us/.

## Ethics statement

The studies involving humans were approved by the McGill University PET Working Committee and the Douglas Mental Institute Research Ethics Board. The studies were conducted in accordance with the local legislation and institutional requirements. The participants provided their written informed consent to participate in this study.

## Author contributions

LS-R: Conceptualization, Data curation, Formal analysis, Investigation, Methodology, Software, Visualization, Writing – original draft, Validation. AK: Resources, Visualization, Writing – review & editing. QA: Resources, Writing – review & editing. GB: Data curation, Writing – review & editing. JT: Data curation, Writing – review & editing. JF-A: Data curation, Writing – review & editing. SS: Data curation, Writing – review & editing. NR: Data curation, Writing – review & editing. CT: Data curation, Writing – review & editing. JS: Data curation, Writing – review & editing. HJ: Resources, Writing – review & editing. XC: Resources, Writing – review & editing. FC: Methodology, Writing – review & editing. PR-N: Funding acquisition, Project administration, Resources, Writing – review & editing. YI-M: Conceptualization, Funding acquisition, Methodology, Project administration, Resources, Supervision, Validation, Writing – review & editing.
