# Supplementary material for: In-vivo neuronal dysfunction by Aβ and tau overlaps with brain-wide inflammatory mechanisms in Alzheimer’s disease
Source: Front Aging Neurosci. 2024 Jun 19;16:1383163. doi: 10.3389/fnagi.2024.1383163 (PMC11223503; doi:10.3389/fnagi.2024.1383163)
Supplement: Supplementary file 1 [file Data_Sheet_1.pdf]

Supplementary Information

In-vivo neuronal dysfunction by Aβ and tau overlaps with brain-wide inflammatory mechanisms in Alzheimer's disease

Lazaro M. Sanchez-Rodriguez[1,2,3], Ahmed F. Khan[1,2,3], Quadri Adewale[1,2,3], Gleb Bezgin[1,2,3,4], Joseph Therriault[1,2,4], Jaime Fernandez-Arias[1,2,4], Stijn Servaes[1,2,4], Nesrine Rahmouni[1,2,4], Cécile Tissot[1,2,4], Jenna Stevenson[1,2,4], Hongxiu Jiang[1,2], Xiaoqian Chai[1,2], Felix Carbonell[5], Pedro Rosa-Neto[1,2,4], Yasser Iturria-Medina[1,2,3*]

[1]Department of Neurology and Neurosurgery, McGill University, Montreal, Canada.

[2]McConnell Brain Imaging Centre, Montreal Neurological Institute, Montreal, Canada.

[3]Ludmer Centre for Neuroinformatics & Mental Health, Montreal, Canada.

[4]McGill University Research Centre for Studies in Aging, Douglas Research Centre, Montreal, Canada.

[5]Biospective Inc., Montreal, Canada.

Supplementary Text 1. Personalized AD neuronal activity model

For each participant, the BOLD signal is generated through coupled differential equations. Firstly, the excitatory and inhibitory firing rates [Daffertshofer and van Wijk, 2011; Gjorgjieva et al., 2016; Wilson and Cowan, 1972] in neural mass k, $E_k(t)$ and $I_k(t)$, are obtained from:

$$\dot{E}_k = \frac{1}{\tau_E}\left[-E_k + S\left(x_{E,k}\right)\right]$$

$$\dot{I}_k = \frac{1}{\tau_I}\left[-I_k + S\left(x_{I,k}\right)\right]$$

$$x_{E,k} = C_{EE}E_k - C_{IE}I_k + P + \frac{\eta}{N}\sum_{l=1,l\neq k}^{N} \mathrm{C}_{lk}E_l$$

$$x_{I,k} = C_{EI}E_k - C_{II}I_k$$

with the sigmoidal activation functions $S_I\left(x_{I,k}\right) = \frac{1}{1+\exp\left[-a_I\left(x_{I,k}-\theta_I\right)\right]} - \frac{1}{1+\exp\left[a_I\theta_I\right]}$ and $S_E\left(x_{E,k}\right) = \frac{1}{1+\exp\left[-a_E\left(x_{E,k}-\theta_{E,k}\right)\right]} - \frac{1}{1+\exp\left[a_E\theta_{E,k}\right]}$. Additionally, each excitatory sigmoidal firing threshold depends on the local amyloid-beta ($A\beta$) and tau loads: $\theta_{E,k} = \theta_0 + \theta_E^{A\beta} \cdot A\beta_k + \theta_E^{Tau} \cdot Tau_k + \theta_E^{A\beta\cdot Tau} \cdot A\beta_k \cdot Tau_k$. This equation is as (1) in the main text, highlighting that the model assumes perturbations to the excitatory parameter by the pathogens. It must be understood that the $A\beta$ and tau accumulations are subject-specific.

The BOLD signal relates to the action potential arriving at the neuronal populations [Logothetis et al., 2001; Sotero and Trujillo-Barreto, 2008; Valdes-Sosa et al., 2009]. All quantities are normalized to baseline values: $\xi_{E,k} = \frac{S_{E,k}}{{S_{E,k}}^0}$ and $\xi_{I,k} = \frac{S_{I,k}}{{S_{I,k}}^0}$, where the superscript denotes values at rest.

Changes in glucose consumption ($g_{E,k}$ and $g_{I,k}$) are linked to the excitatory and inhibitory neuronal inputs in region k. The glucose variables transform into metabolic rates of oxygen for excitatory ($m_{E,k}$) and inhibitory ($m_{I,k}$) activities, and total oxygen consumption (m_k):

$$\dot{g}_{E,k} = z_{E,k}$$

$$\dot{z}_{E,k} = \frac{-2}{\varkappa_E} z_{E,k} - \frac{1}{{\varkappa_E}^2}\left(g_{E,k} - 1\right) + \frac{h_E}{\varkappa_E}\left(\xi_{E,k} - 1\right)$$

$$\dot{g}_{I,k} = z_{I,k}$$

$$\dot{z}_{I,k} = \frac{-2}{\varkappa_I} z_{I,k} - \frac{1}{{\varkappa_I}^2}\left(g_{I,k} - 1\right) + \frac{h_I}{\varkappa_I}\left(\xi_{I,k} - 1\right)$$

$$m_{E,k}(t) = \frac{2 - x(t)}{2 - x_0} g_{E,k}(t)$$

$$m_{I,k}(t) = g_{I,k}(t)$$

$$m_k(t) = \frac{\gamma m_{E,k}(t) + m_{I,k}(t)}{\gamma + 1}$$

$$x(t) = \frac{1}{1 + exp\left[c\left(d - g_{E,k}(t)\right)\right]}$$

Cerebral blood flow (f_k) is modeled as follows [Friston et al., 2000], assuming that CBF is coupled to excitatory activity:

$$\dot{f}_k = y_k$$

$$\dot{y}_k = \frac{-2}{\varkappa_f} y_k - \frac{1}{{\varkappa_f}^2}(f_k - 1) + \mu\left(\xi_{E,k} - 1\right)$$

The outputs of the metabolic and vascular models are converted to normalized cerebral blood volume (b_k) and deoxy-hemoglobin (q_k) content through the Balloon model [Buxton et al., 1998]:

$$\dot{b}_k = \frac{1}{\varkappa_0}(f_k - f_{out})$$

$$\dot{q}_k = \frac{1}{\varkappa_0}\left(m_k - f_{out}\frac{q_k}{b_k}\right)$$

$$f_{out} = b_k^{\frac{1}{\zeta}}$$

The BOLD signal is finally obtained by using the following linear observation equation:

$$BOLD_k(t) = V_0\big(a_1(1 - q_k) - a_2(1 - b_k)\big)$$

where $a_1 = 4.3Y_0E_0 \cdot TE + \varepsilon r_0E_0 \cdot TE$ and $a_2 = \varepsilon r_0E_0 \cdot TE + \varepsilon - 1$ are parameters that depend on the experimental conditions (field strength, TE) [Archila-Meléndez et al., 2020; Deco et al., 2018; Obata et al., 2004; Simon and Buxton, 2015].

Supplementary Text 1—Table 1. Dynamical model parameters*

Parameter	Definition	Value	Ref.
$\begin{bmatrix} \boldsymbol{E_0} \\ \boldsymbol{I_0} \\ g_{E0} \\ z_{E0} \\ g_{I0} \\ z_{I0} \\ f_0 \\ y_0 \\ b_0 \\ q_0 \end{bmatrix}$	Initial conditions	$\begin{bmatrix} 0.075 \\ 0.01 \\ 1 \\ 0 \\ 1 \\ 0 \\ 1 \\ 0 \\ 1 \\ 1 \end{bmatrix}$	[Sotero et al., 2009; Sotero and Trujillo-Barreto, 2007; Sotero and Trujillo-Barreto, 2008; Valdes-Sosa et al., 2009]
τ_I	Time-constant controlling the decay of inhibitory activity after stimulation	0.02 s	[Abeysuriya et al., 2018]
τ_E	Time-constant controlling the decay of excitatory activity after stimulation	0.01 s	[Abeysuriya et al., 2018]
C_{II}	Local inhibitory-inhibitory connection strength	1.2	[Gjorgjieva et al., 2016; Meijer et al., 2015; Wilson and Cowan, 1972]
C_{EI}	Local excitatory-inhibitory connection strength	6	[Gjorgjieva et al., 2016; Meijer et al., 2015; Wilson and Cowan, 1972]
C_{EE}	Local excitatory-excitatory connection strength	6.4	[Gjorgjieva et al., 2016; Meijer et al., 2015; Wilson and Cowan, 1972]
C_{IE}	Local inhibitory-excitatory connection strength	4.8	[Gjorgjieva et al., 2016; Meijer et al., 2015; Wilson and Cowan, 1972]
$\boldsymbol{P}$	Average constant external input received by the excitatory population	0.65 (set to produce plausible simulated electrophysiological and BOLD signals)	[Gjorgjieva et al., 2016; Meijer et al., 2015; Wilson and Cowan, 1972]
a_I	Maximum slope of the inhibitory sigmoidal activation function	1	[Abeysuriya et al., 2018]
a_E	Maximum slope of the excitatory sigmoidal activation function	1	[Abeysuriya et al., 2018]
θ_I	Position of the inhibitory sigmoidal firing function' threshold for activation	4	[Gjorgjieva et al., 2016; Meijer et al., 2015; Wilson and Cowan, 1972]

θ_E	Position of the excitatory sigmoidal firing function' threshold for activation	Variable in [2.75,2.85] depending on the regional pathological loads<br><br>2.8 (in normal baseline conditions)	[Abeysuriya et al., 2018; Daffertshofer and van Wijk, 2011; Gjorgjieva et al., 2016; Meijer et al., 2015; Wilson and Cowan, 1972]
$\boldsymbol{\eta}$	Global coupling strength scaling the anatomical connectivity matrix C_{lk}	2<br>(set to produce plausible simulated electrophysiological and BOLD signals)	[Abeysuriya et al., 2018; Daffertshofer and van Wijk, 2011; Gjorgjieva et al., 2016; Meijer et al., 2015; Wilson and Cowan, 1972]
N	Number of brain regions of interest	66	[Klein and Tourville, 2012]
h_E	Efficacy of glucose consumption response to excitation	1	[Sotero et al., 2009; Sotero and Trujillo-Barreto, 2007; Sotero and Trujillo-Barreto, 2008; Valdes-Sosa et al., 2009]
h_I	Efficacy of glucose consumption response to inhibition	1	[Sotero et al., 2009; Sotero and Trujillo-Barreto, 2007; Sotero and Trujillo-Barreto, 2008; Valdes-Sosa et al., 2009]
$\varkappa_E$	Time-constant of the excitatory glucose consumption impulse response.	1 s	[Sotero et al., 2009; Sotero and Trujillo-Barreto, 2007; Sotero and Trujillo-Barreto, 2008; Valdes-Sosa et al., 2009]
$\varkappa_I$	Time-constant of the inhibitory glucose consumption impulse response.	1 s	[Sotero et al., 2009; Sotero and Trujillo-Barreto, 2007; Sotero and Trujillo-Barreto, 2008; Valdes-Sosa et al., 2009]
c	Steepness of the sigmoid function x	2.5	[Sotero et al., 2009; Sotero and Trujillo-Barreto, 2007; Sotero and Trujillo-Barreto, 2008; Valdes-Sosa et al., 2009]
d	Position of the threshold of the sigmoid function x	1.6	[Sotero et al., 2009; Sotero and Trujillo-Barreto, 2007; Sotero and Trujillo-Barreto, 2008; Valdes-Sosa et al., 2009]
γ	Baseline ratio of excitatory to inhibitory synaptic activity in the voxel	5	[Sotero et al., 2009; Sotero and Trujillo-Barreto, 2007; Sotero and Trujillo-Barreto, 2008; Valdes-Sosa et al., 2009]

x_0	Fraction of glucose following the glycogenolitic pathway at rest	$\frac{1}{1+exp\left[c\left(d-1(t)\right)\right]}$	[Sotero et al., 2009; Sotero and Trujillo-Barreto, 2007; Sotero and Trujillo-Barreto, 2008; Valdes-Sosa et al., 2009]
μ	Efficacy of blood flow response to excitation	0.8	[Sotero et al., 2009; Sotero and Trujillo-Barreto, 2007; Sotero and Trujillo-Barreto, 2008; Valdes-Sosa et al., 2009]
$\varkappa_f$	Time constant for CBF response	1.7	[Sotero et al., 2009; Sotero and Trujillo-Barreto, 2007; Sotero and Trujillo-Barreto, 2008; Valdes-Sosa et al., 2009]
$\varkappa_0$	Transit time through the balloon	1	[Sotero et al., 2009; Sotero and Trujillo-Barreto, 2007; Sotero and Trujillo-Barreto, 2008; Valdes-Sosa et al., 2009]
ζ	Coefficient of the steady state flow-volume relationship	0.4	[Sotero et al., 2009; Sotero and Trujillo-Barreto, 2007; Sotero and Trujillo-Barreto, 2008; Valdes-Sosa et al., 2009]
V_0	Baseline blood volume	0.03	[Sotero et al., 2009; Sotero and Trujillo-Barreto, 2007; Sotero and Trujillo-Barreto, 2008; Valdes-Sosa et al., 2009]
Υ_0	frequency offset of a fully deoxygenated blood vessel at 3 T	80.6 s^{-1} (at 3 T)	[Archila-Meléndez et al., 2020; Obata et al., 2004; Simon and Buxton, 2015]
r_0	Slope defining the dependence of the R2* relaxation rate on blood oxygenation	178 s^{-1} (at 3 T)	[Archila-Meléndez et al., 2020; Obata et al., 2004; Simon and Buxton, 2015]
E_0	Baseline oxygen extraction fraction	0.4	[Archila-Meléndez et al., 2020; Obata et al., 2004; Simon and Buxton, 2015]
ε	Intrinsic ratio of blood to tissue signals at rest	0.24	[Archila-Meléndez et al., 2020; Obata et al., 2004; Simon and Buxton, 2015]
TE	Echo time	32.0 ms	https://triad.tnl-mcgill.com/

*All parameters except θ_E were set to the values indicated in the table for all brain regions and subjects. The values of parameters in **bold** letters were chosen following a dynamical bifurcation analysis of the isolated Wilson-Cowan oscillator and a global analysis of the possible BOLD signals that were generated in the connected model for the θ_E interval (e.g., η values producing highly regular and unrealistic BOLD signals were dropped) [Daffertshofer and van Wijk, 2011; Deco and Martí, 2007; Stefanovski et al., 2019]. θ_E varies according to equation (1) in the main text, and its Aβ-, tau- and Aβ·tau- components were individually estimated for each participant.

References

Abeysuriya RG, Hadida J, Sotiropoulos SN, Jbabdi S, Becker R, Hunt BAE, Brookes MJ, Woolrich W (2018): A biophysical model of dynamic balancing of excitation and inhibition in fast oscillatory large-scale networks.

Archila-Meléndez ME, Sorg C, Preibisch C (2020): Modeling the impact of neurovascular coupling impairments on BOLD-based functional connectivity at rest. Neuroimage 218.

Buxton RB, Wong EC, Frank LR (1998): Dynamics of blood flow and oxygenation changes during brain activation: The balloon model. Magn Reson Med 39:855–864.

Daffertshofer A, van Wijk BCM (2011): On the Influence of Amplitude on the Connectivity between Phases. Front Neuroinform 5:6. http://journal.frontiersin.org/article/10.3389/fninf.2011.00006/abstract.

Deco G, Cruzat J, Cabral J, Knudsen GM, Carhart-Harris RL, Whybrow PC, Logothetis NK, Kringelbach ML (2018): Whole-Brain Multimodal Neuroimaging Model Using Serotonin Receptor Maps Explains Non-linear Functional Effects of LSD. Current Biology 28:3065-3074.e6. https://linkinghub.elsevier.com/retrieve/pii/S0960982218310455.

Deco G, Martí D (2007): Deterministic analysis of stochastic bifurcations in multi-stable neurodynamical systems. Biol Cybern 96:487–96. http://www.ncbi.nlm.nih.gov/pubmed/17387505.

Friston KJ, Mechelli A, Turner R, Price CJ (2000): Nonlinear responses in fMRI: The balloon model, Volterra kernels, and other hemodynamics. Neuroimage 12:466–477.

Gjorgjieva J, Evers JF, Eglen SJ (2016): Homeostatic activity-dependent tuning of recurrent networks for robust propagation of activity. Journal of Neuroscience 36:3722–3734.

Klein A, Tourville J (2012): 101 Labeled Brain Images and a Consistent Human Cortical Labeling Protocol. Front Neurosci 6:1–12.

Logothetis NK, Pauls J, Augath M, Trinath T, Oeltermann A (2001): Neurophysiological investigation of the basis of the fMRI signal.

Meijer HGE, Eissa TL, Kiewiet B, Neuman JF, Schevon CA, Emerson RG, Goodman RR, McKhann GM, Marcuccilli CJ, Tryba AK, Cowan JD, van Gils SA, van Drongelen W (2015): Modeling focal epileptic activity in the Wilson-cowan model with depolarization block. J Math Neurosci 5:7. http://www.pubmedcentral.nih.gov/articlerender.fcgi?artid=4385301&tool=pmcentrez&rendertype=abstract.

Obata T, Liu TT, Miller KL, Luh W, Wong EC, Frank LR, Buxton RB (2004): Discrepancies between BOLD and flow dynamics in primary and supplementary motor areas : application of the balloon model to the interpretation of BOLD transients 21:144–153.

Simon AB, Buxton RB (2015): Understanding the dynamic relationship between cerebral blood flow and the BOLD signal: Implications for quantitative functional MRI. Neuroimage 116:158–167. http://dx.doi.org/10.1016/j.neuroimage.2015.03.080.

Sotero RC, Trujillo-Barreto NJ (2007): Modelling the role of excitatory and inhibitory neuronal activity in the generation of the BOLD signal. Neuroimage 35:149–165. http://dx.doi.org/10.1016/j.neuroimage.2006.10.027.

Sotero RC, Trujillo-Barreto NJ (2008): Biophysical model for integrating neuronal activity, EEG, fMRI and metabolism. Neuroimage 39:290–309.

Sotero RC, Trujillo-Barreto NJ, Jiménez JC, Carbonell F, Rodríguez-Rojas R (2009): Identification and comparison of stochastic metabolic/hemodynamic models (sMHM) for the generation of the BOLD signal. J Comput Neurosci 26:251–69. http://www.ncbi.nlm.nih.gov/pubmed/18836824.

Stefanovski L, Triebkorn P, Spiegler A, Diaz-Cortes MA, Solodkin A, Jirsa V, McIntosh AR, Ritter P (2019): Linking Molecular Pathways and Large-Scale Computational Modeling to Assess Candidate Disease Mechanisms and Pharmacodynamics in Alzheimer's Disease. Front Comput Neurosci 13:1–27.

Valdes-Sosa PA, Sanchez-Bornot JM, Sotero RC, Iturria-Medina Y, Aleman-Gomez Y, Bosch-Bayard J, Carbonell F, Ozaki T (2009): Model driven EEG/fMRI fusion of brain oscillations. Hum Brain Mapp 30:2701–2721.

Wilson HR, Cowan JD (1972): Excitatory and inhibitory interactions in localized populations of model neurons. Biophys J 12:1–24. http://www.sciencedirect.com/science/article/pii/S0006349572860685.

Supplementary Table 1. Demographics of the samples

	CU	AD	p
Number of individuals (N)	47	16	-
Age (yrs), mean (s.d.)	68.84 (8.36)	69.45 (9.10)	0.801
Female, N (%)	36 (76.6)	8 (50.0)	0.061
Education (yrs), mean (s.d.)	15.64 (3.59)	14.31 (3.38)	0.210
APOE ε4 carriers, N (%)	11 (23.4)	7 (43.8)	0.198
MMSE, mean (s.d.)	29.33 (0.88)	20.25 (7.20)	< 0.001
Aβ+, N (%)	0 (0)	16 (100.0)	< 0.001
Braak > 0, N (%)	0 (0)	16 (100.0)	< 0.001

P-values for age, education and MMSE indicate values assessed with two-sided independent-samples t-tests. For the resting variables (sex, *APOE* ε4 status, Aβ+ and Braak > 0), Fischer exact tests were performed. CU cognitively unimpaired; AD Alzheimer's disease; *APOE* ε4, apolipoprotein epsilon 4; MMSE, Mini-Mental State examination.

Supplementary Table 4. Genes with most appearances (%) in the top statistically significant biological pathways identified for the Aβ+tau → neuronal-activity gene list.

Gene	%	Gene	%	Gene	%	Gene	%	Gene	%
RIPK2	45.7	*CLEC7A*	21.7	*TICAM2*	17.2	*SLAMF6*	14.5	*C5AR1*	12.7
SYK	45.2	*TYROBP*	21.7	*RPH3AL*	17.2	*TLR6*	14.5	*OSR1*	12.2
ANXA1	41.2	*SNCA*	21.3	*MAPK1*	17.2	*VAMP8*	14.5	*HIPK2*	12.2
IL12B	38.0	*NAGLU*	21.3	*ITPKB*	17.2	*SRF*	14.5	*CD84*	12.2
CCL19	36.2	*CD1D*	21.3	*HLA-DRA*	17.2	*PTCH1*	14.5	*FCN3*	12.2
HLA-DRB1	35.7	*ZBTB16*	20.8	*CD38*	17.2	*PLA2G5*	14.5	*NCK2*	12.2
CCL5	33.0	*VAV1*	20.8	*GAL*	16.7	*HLA-DPB1*	14.5	*TNFRSF4*	12.2
IHH	32.6	*B2M*	20.4	*BMP7*	16.7	*CCR1*	14.5	*TLR1*	12.2
PTPRC	31.7	*WNT10B*	19.9	*SPHK2*	16.3	*TBX3*	14.0	*SKI*	12.2
CD74	31.7	*IL2RG*	19.9	*PLCB1*	16.3	*OPRK1*	14.0	*PIK3R1*	12.2
IL1B	31.2	*GRP*	19.9	*CD160*	16.3	*CRHBP*	14.0	*HLA-DPA1*	12.2
IL18	30.8	*F2*	19.9	*EBI3*	16.3	*AIF1*	14.0	*MTOR*	12.2
TGFB1	29.9	*EPO*	19.9	*SOX11*	16.3	*FCRL3*	13.6	*CPLX1*	11.8
F2RL1	29.9	*NF1*	19.5	*PIK3CG*	16.3	*CYP26B1*	13.6	*PAX2*	11.8
BMP4	29.9	*KITLG*	19.5	*NPY*	16.3	*BAIAP3*	13.6	*GNAI2*	11.8
PYCARD	29.4	*FYN*	19.5	*ITGAM*	16.3	*SOD1*	13.6	*APLNR*	11.8
PLCG2	28.5	*GHRL*	19.0	*IL1A*	16.3	*PTGER4*	13.6	*SYT2*	11.3
NCKAP1L	26.7	*GPR68*	19.0	*CD36*	16.3	*NTSR1*	13.6	*CADPS2*	11.3
EDN1	26.2	*CCL2*	19.0	*CBFB*	16.3	*CRH*	13.6	*SYT12*	11.3
CD80	26.2	*PTAFR*	19.0	*OPRM1*	15.8	*BBS4*	13.6	*RPH3A*	11.3
CD86	25.8	*CFTR*	19.0	*C3*	15.8	*TLR7*	13.1	*FGL2*	11.3
HLA-G	25.3	*RBP4*	18.6	*PELI1*	15.4	*MBL2*	13.1	*PDE5A*	11.3
HAVCR2	24.9	*INS*	18.6	*CITED2*	15.4	*ADCYAP1*	13.1	*EOMES*	11.3
SOX4	24.9	*LILRB2*	18.1	*KIAA0748*	15.4	*TNIP2*	12.7	*LAPTM5*	11.3
WNT3A	24.4	*STXBP1*	18.1	*RAG1*	15.4	*CPLX2*	12.7	*SPINK1*	11.3
HLA-E	24.4	*GPR183*	18.1	*LRP5*	15.4	*NRP1*	12.7	*PSMD9*	11.3
WNT7A	24.0	*IRF4*	17.6	*GATA2*	15.4	*KCNQ1*	12.7	*NOS2*	11.3
KIT	24.0	*HLA-F*	17.6	*ACTL6A*	15.4	*IL12A*	12.7	*CHGA*	11.3
CARD11	23.1	*FGB*	17.6	*STXBP3*	14.9	*CD70*	12.7	*CEBPA*	11.3

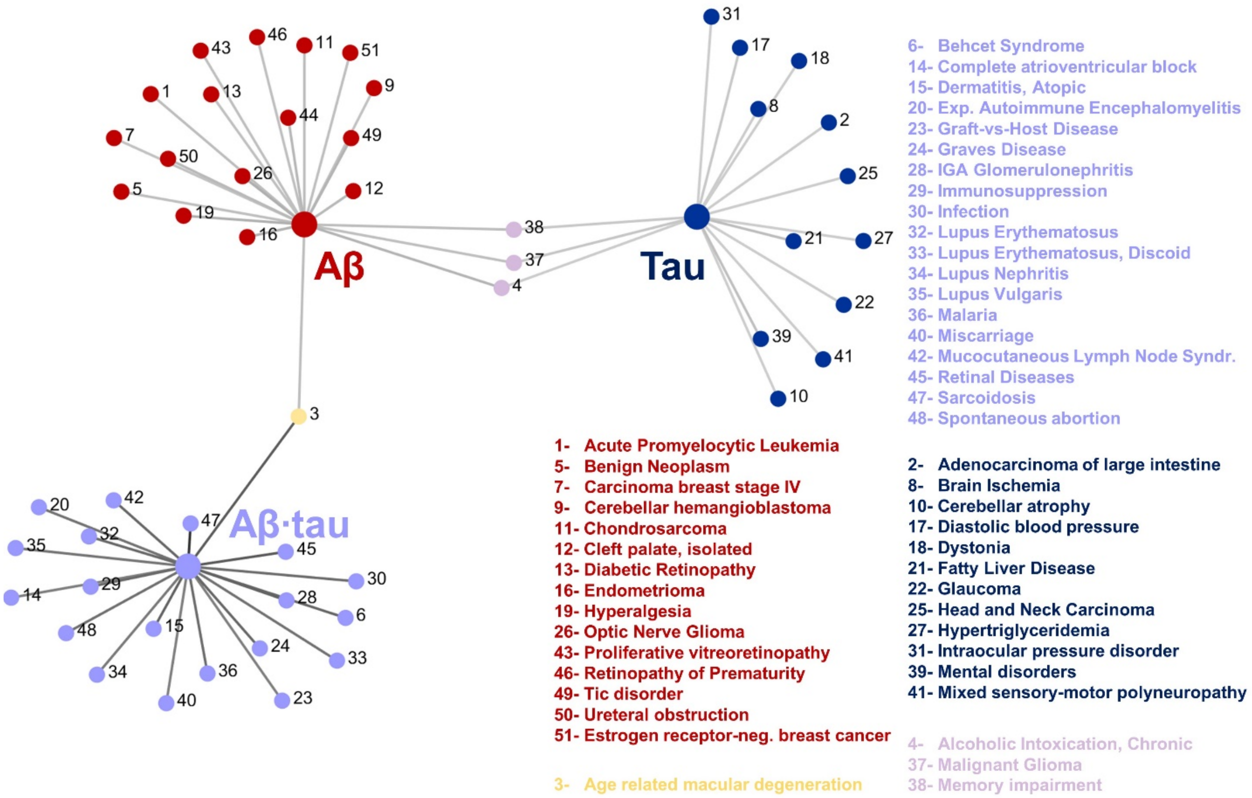

Supplementary Figure 1. AD associated diseasome. To visually represent the associations between the AD Aβ, tau and Aβ·tau molecular associates and other disease-characteristic molecular pathways, the grayscale color of a link in the network plot is inversely proportional to the q-value of the enrichment statistical test, i.e., darker edges reflect increased statistical significance that the dysfunctional disease pathways are overrepresented in the corresponding gene set. Only significantly enriched terms are shown (hypergeometric tests, $q < 0.05$, Benjamini-Hochberg corrected), to a maximum of 20 terms. Top disease pathways that were shared by the functional genetic signatures connect the Aβ, tau and Aβ·tau clusters, being represented with additional colors. The network plot was annotated on the right with the names of the associated pathologies for increased readability. The disease pathways are curated in DisGeNET and were accessed through Metascape (Piñero et al., 2017; Zhou et al., 2019).

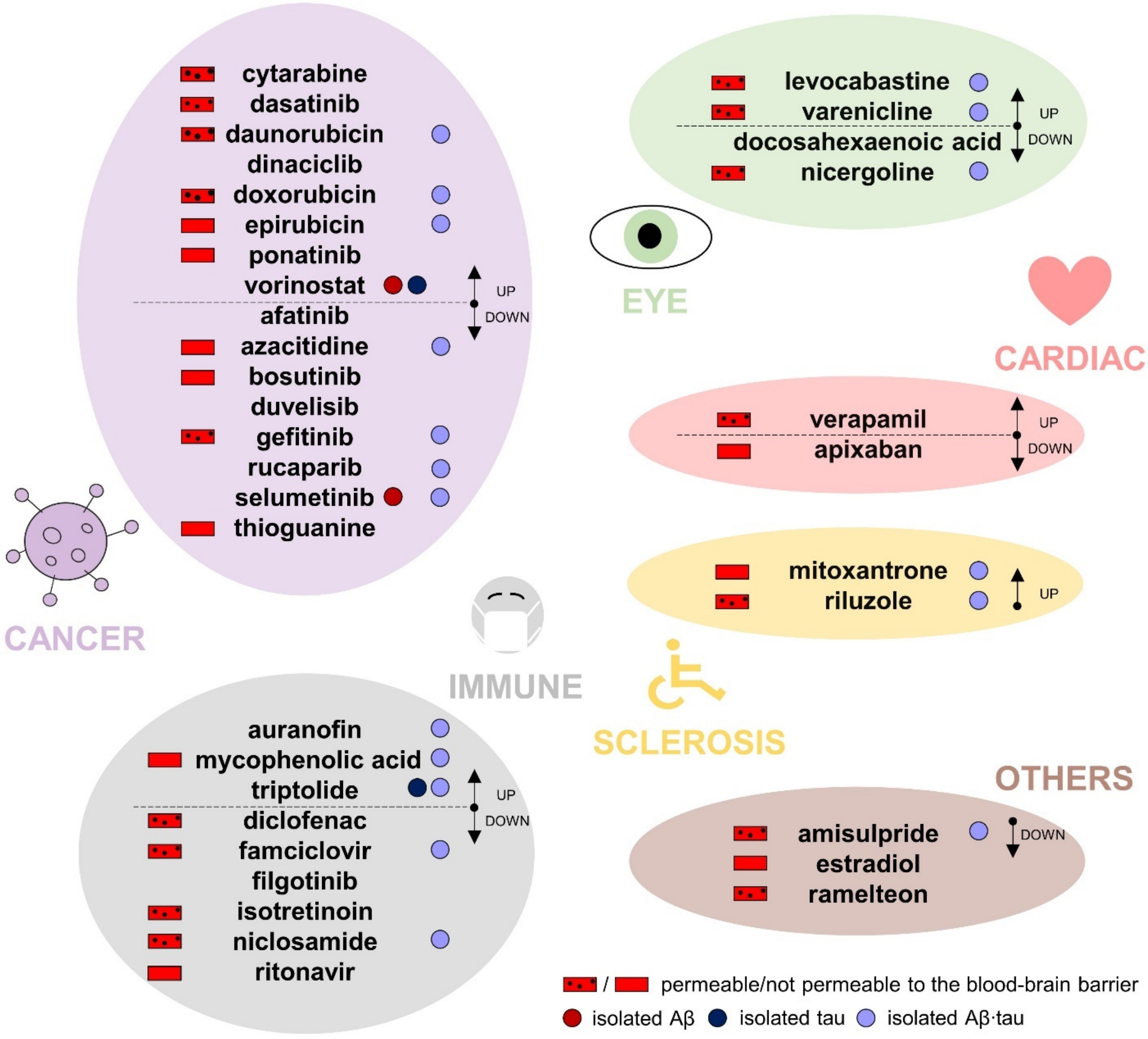

Supplementary Figure 2. Top identified drug repurposing candidates to target adverse Aβ- and tau-induced neuronal-activity effects. Reported are existing drugs which molecular interactions would induce gene expression changes in the set of all Aβ-, tau- and Aβ·tau-associated genes (Mann–Whitney U test, $q < 0.05$, Benjamini-Hochberg corrected). The predicted chemical compounds have been organized in major groups according to their drug use indications. All medications are FDA-cleared for either treatment of cancer, various immune system/infection/inflammatory processes (“immune”), eye diseases, cardiovascular conditions, multiple or amyotrophic lateral sclerosis or “other” disorders. The groups are further divided by

whether the candidate drug up- or down-regulates the genes linked to the neuronal activity alterations by AD. Additionally, blood-brain barrier permeability, when this information was available, is specified next to the name of the drug (red rectangles on the left). Drugs that could also target the separate Aβ, tau or Aβ·tau molecular associates are identified with accordingly colored circles on the right of the compound's name, e.g., the chemical selumetinib may be used to modify Aβ- and Aβ·tau- associated gene sets.

Supplementary Pseudocode 1.

```
// program to calculate the personalized combined neuronal activity influences by Aβ, tau and
// Aβ · Tau

{       // definitions
Define surrogate optimization parameters (bounds and constraints, number of iterations)
Load the subject's Aβ, tau and fALFF (rs-fMRI) and anatomical connectivity matrix
Define the neuronal activity influence model (Eq. 1)
Define neural mass model and transformations to simulate the resting-state BOLD signal
Define the objective function (minimize correlation distance between real and simulated BOLD)
}

{       // optimization
FOR i = 1 TO 20        // different random optimization evaluation trials
        Perform surrogate optimization until the algorithm converges
                // At each iteration:
                // simulate the BOLD signal,
                // calculate similarity with the subject's real signal,
                // retain the best evaluation thus far
                // (performed by Matlab's surrogateopt.m)
        Save the optimized neuronal activity affectation parameters and optimization outputs
ENDFOR
}

{       // post-processing
Retain the optimization outcome with the lowest overall cost
Reconstruct hidden quantities of interest, e.g., neuronal excitabilities, spectral power, etc
}
```
